# Effects of heel apex position, apex angle and rocker radius on plantar pressure in the heel region

**DOI:** 10.1016/j.heliyon.2023.e21036

**Published:** 2023-10-16

**Authors:** Athra Malki, Laurens van Kouwenhove, Gijsbertus J. Verkerke, Rienk Dekker, Juha M. Hijmans

**Affiliations:** a Department of Rehabilitation Medicine, University of Groningen, University Medical Center Groningen, Groningen, the Netherlands; b Department of Biomechanical Engineering, University of Twente, Enschede, Overijssel, the Netherlands

**Keywords:** Diabetes mellitus, Footwear, Rocker shoe, Heel pressure, Diabetic foot ulcer, Plantar pressure, Diabetic foot, Prevention

## Abstract

**Introduction:**

Rocker shoes and insoles are used to prevent diabetic foot ulcers in persons with diabetes mellitus and loss of protective sensation, by reducing the plantar pressure in regions with high pressure values (>200 kPa) (e.g., hallux, metatarsal heads and heel). However, forefoot rocker shoes that reduce pressure in the forefoot inadvertently increase pressure in the heel. No studies focused on mitigating the negative effects on heel pressure by optimizing the heel rocker midsole, yet. Therefore, we analyze the effect of different heel rocker parameters on the heel plantar pressure.

**Methods:**

In-shoe pressure was measured, while 10 healthy participants walked with control shoe and 10 different heel rocker settings. Peak pressure was determined in 7 heel masks, for all shoes. Generalized estimating equations was performed to test the effect of the different shoes on the peak pressure in the different heel masks.

**Results:**

In the proximal heel, a rocker shoe with distal apex position, small rocker radius and large apex angle (100°), shows the largest significant decrease in peak pressure compared to rocker shoes with more proximally located apex positions. In the midheel and distal heel, the same rocker shoes or any other rocker shoes, analyzed in this study, do not reduce the PP more than 2 % compared to the control shoe. For the midheel and distal heel region with high pressure values (>200 kPa), rocker shoes alone are not the correct option to reduce the pressure to below 200 kPa.

**Conclusion:**

When using rocker shoes to reduce the pressure in the forefoot, a heel rocker midsole with a distal apex position, small rocker radius and apex angle of 100°, mitigates the negative effects on proximal heel pressure. For the midheel and distal heel, other footwear options as an addition or instead of rocker shoes are needed to reduce the pressure.

## Highlights

• Relation between heel rocker parameters and heel pressure was studied.
• Distal apex, small radius & angle (100°) mitigate pressure rise in proximal heel.
• No rocker shoe reduced pressure >2 % compared to control shoe in mid & distal heel.
• Use other options to reduce pressure in mid & distal heel combined/without rockers.

## 1 Introduction

Diabetes Mellitus (DM) is a worldwide health issue affecting 537 million people between the ages 20–79 years [1]. Persons with DM often suffer from diabetic foot ulcers (DFUs) [2]. The hallux, metatarsal heads (MTH) and the heel are considered to be high risk regions in developing DFUs [3]. Ulcers in the heel occur less frequently than the ones in the forefoot, however they are more difficult and 1.5 times more expensive to treat [4]. Repetitive stress on the heel, in combination with loss of protective sensation (LOPS) due to peripheral neuropathy and loss of fat pad quality due to DM, leads to unnoticed skin breakdown that often results in DFUs [[5], [6], [7]].

In order to prevent DFUs from happening, high plantar pressure values need to be reduced. According to the International Working Group on the Diabetic Foot (IWGDF) guidelines on the prevention and management of the diabetic foot, plantar pressure values of 200 kPa and above are considered to be a risk for developing DFUs and need to be reduced to below 200 kPa or by at least 30 % [8]. Plantar pressure is a result of the interaction between the ground reaction force (GRF) and the plantar surface of the foot (pressure = force/contact area). In order to reduce the pressure, either the force must be reduced (or shifted to an area with low pressure) or the plantar surface area of the foot must be increased. This can be achieved by insoles that increase the contact area between the insole and the foot or by custom-made rocker shoes [9]. Rocker shoes aim to reduce the pressure by redirecting the point of application of the GRF away from regions with high pressure to regions with low pressure. This can be achieved by creating a curved rigid rocker shoe midsole, designed to rock the foot from heel strike to toe-off, while restricting the motion of the joints in the foot [[10], [11], [12], [13]]. The rocker midsole can be altered by manipulation of the rocker shoe parameters (e.g., changing the orientation and position of the apex), in order to redirect the point of application of the GRF, to achieve proper pressure redistribution [[11], [12], [13], [14], [15], [16], [17]].

**Fig. 1:**
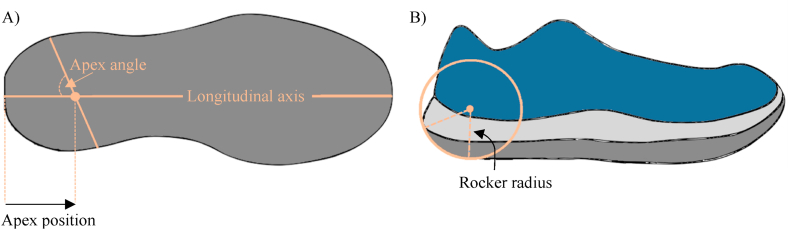
Heel rocker parameters for the rocker shoe. A: apex position and apex angle. B: rocker radius at the apex position.

The rocker parameters that characterize the shape of rocker shoes are the apex position, apex angle and rocker radius for both heel and forefoot (see Fig. 1A and B for heel rocker parameters). The heel apex position is the point on the longitudinal axis where the rocker ends. The heel apex angle is the angle between the apex and longitudinal axis of the shoe. The heel rocker radius is the curvature of the rocker. Several studies have examined methods to reduce high peak pressure (PP) in the forefoot region by altering forefoot rocker shoe design features [[11], [12], [13], [14], [15], [16], [17]]. Placing the apex position between 50 % and 60 % of the longitudinal axis for instance, reduces the PP in the MTH region while an apex position of 65 % reduces the PP in the hallux region [11,[13], [14], [15], [16], [17]]. Apex angles between 90° and 100° reduce the PP in MTH1 and hallux and between 70°and 80° in MTH5 [13,17]. Regarding the forefoot rocker radius, a small radius (62 % shoe length) reduces the PP in the hallux and other toes, while a small to medial (71 % of shoe length) rocker radius reduces the PP in the distal heel [14]. Although the biomechanical effect of the forefoot rocker parameters is known and plantar pressure redistribution can be achieved in the forefoot, the same interventions tend to increase the PP in the heel region [11,13,14,16,17].

As rocker shoes can increase the PP in the heel, when used as an intervention to decrease the PP in the forefoot, it is crucial to understand the optimal selection of heel rocker parameters to at least mitigate the PP increase in the heel. However, to our knowledge, the effect of the heel rocker parameters on the heel plantar pressure has not been studied yet [13,14,17]. Therefore, there is a need for more knowledge on the role of different heel rocker parameters on the pressure redistribution in the heel region [13,14,17]. Once there is more knowledge on the effect of heel rocker parameters in reducing high pressure values, a combination of parameters for the forefoot rocker shoe and a heel rocker could be integrated in one shoe to reduce the pressure across the entire foot (if required). Hence, this study aims to analyze the effect of different heel rocker parameters on the heel plantar pressure.

In line with the forefoot, we expect the pressure to be redistributed towards the apex position, therefore, we hypothesize that the pressure will be increased at the apex position and reduced in regions located both proximal and distal to the apex position [11,[13], [14], [15], [16], [17]]. Also in line with the forefoot rocker mechanism, we expect a mediolateral (ML) shift of pressure with a change in apex angle, where a larger angle will result in lower pressure in the lateral heel region and a smaller angle in the medial heel region [13,17]. In contrast to the forefoot rocker, we expect that smaller radii will increase the pressure in the region distal to the apex, while a larger rocker radius will increase the pressure in the region proximal to the apex [14].

## 2 Methods

### 2.1 Participants

Ten healthy individuals participated in this pilot study. The sample size was based on prior experience with studies on reducing plantar pressure with therapeutic footwear [14,18]. The inclusion criteria were: healthy adults between the ages of 18–30 years who had shoe size of EU41 (only one size of the adjustable shoes was made). The exclusion criteria were: the use of walking aids during gait, self-reported injuries or diseases that influence gait. Prior to starting the experiments and the data collection, all participants were informed about the purpose of the study and provided written informed consent. The Medical Ethics Review Board of the University Medical Center Groningen (UMCG) declared that the study falls outside the Dutch law on ‘Medical Research involving Human Subjects’ (WMO). Therefore, no formal approval was required from the Committee (METc 2018/060).

### 2.2 Shoe design

**Fig. 2:**
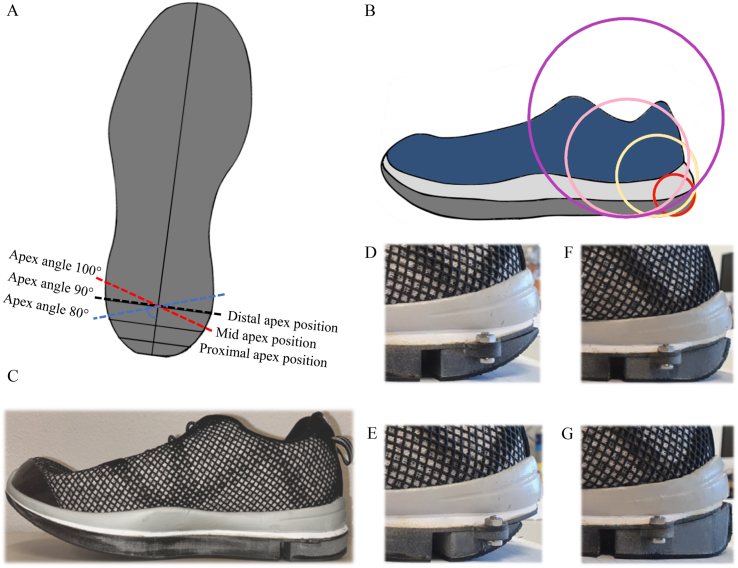
The different heel apex angles are represented by the dotted lines in figure A: the red dotted line: apex angle of 100°, the black dotted line: apex angle of 90° and blue dotted line: apex angle of 80°. The distal (20 % of shoe length, referenced to the heel), mid (11 % of shoe length, referenced to the heel) and proximal (6 % of shoe length, referenced to the heel) heel apex positions are also shown in figure A. Figure B illustrates the rocker shoe with the large heel radius, 22 % of shoe length with an apex position of 20 % of shoe length (purple circle). The same figure shows the small heel radii, 14 % with an apex position of 20 % (pink circle), 10 % with an apex position of 11 % (yellow circle) and 6 % with an apex position of 6 % of shoe length (red circle). Figure C represents the real image of the control shoe and figure D (purple circle in figure B), E (pink circle), F (yellow circle), and G (red circle) represent the heel rocker shoes that are schematically represented in figure B.

A lightweight medium width athletic shoes (Jason, Dr Comfort, Mequon, WI, USA) size EU41 (shoe length 28.8 cm) was used as experimental and control shoe. The midsole of the experimental shoe was replaced with a forefoot rocker midsole, to which different heel parts could be attached. These heel parts had different configurations of the apex position, apex angle and rocker radius. Three apex positions were used, proximal, mid and distal (respectively at 6 %, 11 % and 20 % of the shoe length, referenced to the heel). In line with the forefoot, we wanted to test whether the pressure is reduced in regions located distal and proximal to the apex position and increased at the apex position [11,[13], [14], [15], [16], [17]]. We opted for the distal apex position to be located at the ankle joint, the mid apex position to be located halfway the ankle joint and the heel, and the proximal apex position positioned halfway between the mid apex position and the heel. Three different apex angles (100°, 90° and 80°) were used at each apex position. In line with the forefoot, we wanted to test the effect of a neutral angle, which equates to 90°, and also analyze whether changing the angle to a smaller (80°) or larger (100°) value would cause any mediolateral PP shift [13,17]. Lastly, at the distal apex position two rocker radii were used. The radii at the distal apex position were 4.0 cm (small radius, 14 % shoe length) and 6.3 cm (large radius, 22 % shoe length). At the mid apex position, the radius was 2.8 cm (10 % shoe length), at the proximal apex position, 1.6 cm (6 % shoe length). In line with the forefoot, we wanted to test whether large and/or small radii have an effect on the PP in the heel [14]. As there were no prior tests or specific recommendations for heel rocker radii, we selected a larger radius, approximately matching the distance from the apex position to the ankle joint axis. The combination of configurations of the heel rocker parameters and the control shoe are shown in Fig. 2A–G and Table 1. The materials used for the rocker midsoles are: A thin (5 mm) layer of Ethyl Vinyl Acetate (Shore 63A) and a 13 mm thick stiff sole (3D printed Polyamide 12; Shore 80D). The outsole consisted of a 2 mm rubber material to add grip during walking. All shoes had the same insole (Shore 25A, thickness: 6 mm).

**Table 1:** The control shoe and 10 rocker shoes, configurated with different heel apex positions (20 %, 11 % and 6 %), apex angles (100°, 90° and 80°) and rocker radii (small: 14 %, 10 %, 6 % and large: 22 %).

Condition	Abbreviation of the condition	Apex position (% of shoe length)	Apex angle (°)	Rocker radius (% of shoe length, small/large)
1	AP20A100	20	100	14 (small)
2	AP11A100	11	100	10 (small)
3	AP6A100	6	100	6 (small)
4	AP20A90RSmall	20	90	14 (small)
5	AP20A90RLarge	20	90	22 (large)
6	AP11A90	11	90	10 (small)
7	AP6A90	6	90	6 (small)
8	AP20A80	20	80	14 (small)
9	AP11A80	11	80	10 (small)
10	AP6A80	6	80	6 (small)
11	Control	–	–	–

The numbers 1–11 represent the 10 different rocker shoes and the control shoe.

The abbreviations represent the different rocker shoes: AP20A100, AP11A100, AP6A100, AP20A90RSmall, AP20A90RLarge, AP11A90, AP6A90, AP20A80, AP11A80 and AP6A80.

### 2.3 Experimental procedure

The experimental procedure was performed at the Motion Lab of the Department of Rehabilitation Medicine, University Medical Center Groningen. Height and bodyweight were determined prior to starting the experiment. The pressure was measured with the Pedar-X insoles, placed in the (rocker) shoes (Novel; Munich, Germany, *Fs* = 100 Hz). All participants started the measurements with the control shoe in order to determine the preferred walking speed during that trial. The walking speed of the participants was recorded with a ten camera Vicon system (Vicon Bonita 10, Oxford, UK; *Fs* = 100 Hz). Marker trajectories of 4 reflective markers on the pelvic (right and left anterior and posterior superior iliac spine) were used to determine the preferred walking speed. The walking speed of the trials that followed afterwards had to be within a range of ±5 %. The measurements with the rocker shoes followed in a random order. Three trials with in-shoe pressure measurements were done with each rocker shoe. Participants had to walk a distance of 10 m per trial.

### 2.4 Data analysis

**Fig. 3:**
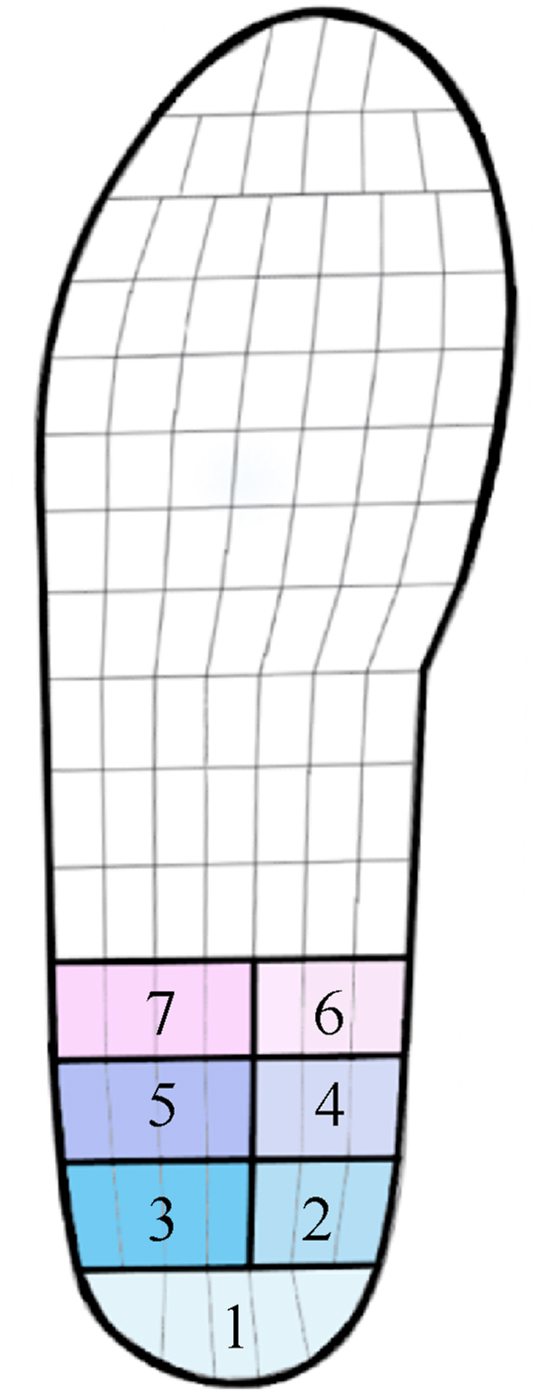
Heel masks of the Pedar-X insole. The numbers represent the following masks, 1: proximal central heel region, 2: medial side of proximal heel region, 3: lateral side of proximal heel region, 4: medial side of midheel region, 5: lateral side of midheel region, 6: medial side of distal heel region, 7: lateral side of distal heel region.

For three trials, four midgait steps of the right foot were selected using Pedar-X Step analysis (Novel; Munich, Germany), resulting in a total of twelve steps per rocker shoe. Twelve steps per foot were measured for reliable and valid in-shoe plantar pressure data [19]. The sensors of the Pedar-X insoles are often grouped in 1 heel mask (Pedar-X sensor 1–26) [[20], [21], [22], [23]], however for the purpose of this paper the heel region was divided in seven different heel masks (Fig. 3). This division is done with the purpose to analyze whether altering rocker parameters (proximal, mid and distal apex position; small and large radii; small, neutral and large apex angle) have distinct effects on a specific heel region. Should such distinctions emerge, this knowledge could be applied in future studies, allowing researchers to adopt an alternative mask division for targeted offloading in the heel. The twelve steps per heel mask were further analyzed in Matlab (R2017b). The PP was calculated within each mask by determining the largest pressure for each step per mask and calculating the average across all the twelve steps.

### 2.5 Statistical analysis

Means and standard deviations were calculated to describe the study population and outcomes of the plantar pressure measurements. A generalized estimating equations (GEE) was conducted using an exchangeable working correlation matrix with the shoe condition as within-subjects variable and PP as dependent variable. For pairwise comparison using GEE, a Bonferroni correction for 10 experimental conditions was applied, resulting in a level of significance set at *P* < 0.001. All statistical analyses were performed using SPSS statistics (26.0).

## 3 Results

### 3.1 Study population characteristics

The study population, consisting of 10 participants (2 male and 8 female), had a mean (±SD) age of 20.7 (±1.3) years, body height of 1.81 (±0.06) m and bodyweight of 65.6 (±7.3) kg. The mean comfortable waking speed was 1.34 (±0.14) m/s.

### 3.2 Effect of the different rocker shoe designs on peak plantar pressure in different heel regions

**Fig. 4:**
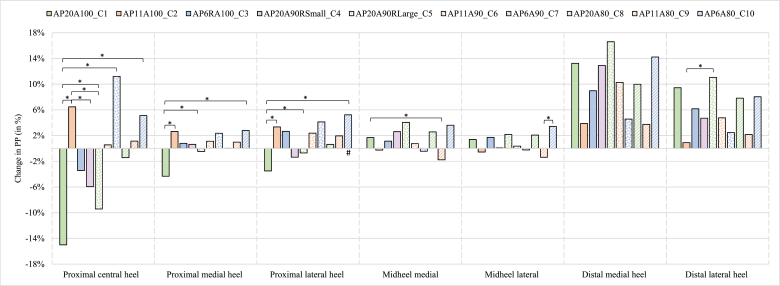
Relative changes in peak pressure (compared to the control condition) for different conditions based on the combination of different heel apex positions (20 %, 11 % and 6 %), apex angles (100°, 90° and 80°) and rocker radii (small: 14 %, 10 %, 6 % and large: 22 %), with the following abbreviations for the different conditions: AP20A100_C1, AP11A100_C2, AP6A100_C3, AP20A90RSmall_C4, AP20A90RLarge_C5, AP11A90_C6, AP6A90_C7, AP20A80_C8, AP11A80_C9 and AP6A80_C10. Negative percentages indicate a decrease in peak pressure compared to the control condition, while positive percentages show an increase. *#:* Significant difference compared to control (*P <* 0.001). *: Significant difference between conditions (*P* < 0.001).

The means and standard deviations of the PP for each condition per mask are shown in Table 2 and the relative changes between the conditions are presented in Fig. 4. The GEE showed a significant difference between the shoe conditions in PP for different heel masks. The statistically significant differences in the pairwise comparisons for PP per condition for each mask are further outlined below and also presented in Fig. 4.

**Table 2:**
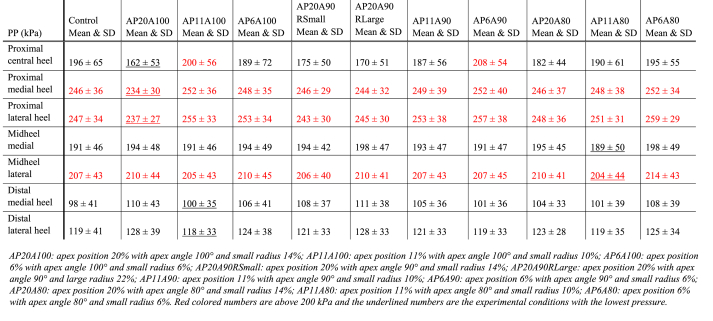
Absolute peak pressure (PP) values for different conditions in seven heel masks.

### 3.3 Proximal central heel

For the proximal central heel region (mask 1), the condition with the largest reduction in PP is AP20A100 (abbreviations of the conditions with the corresponding heel rocker parameters is given in Table 2). This condition (AP20A100) shows a significant decrease in PP compared to the conditions AP20A90RLarge (*P* = 0.004), AP11A100 (*P* < 0.001), AP6A90 (*P* < 0.001), AP11A80 (*P* = 0.032) and AP6A80 (*P* < 0.001).

#### 3.3.1 Medial and lateral side of proximal heel

For the proximal heel region on the medial side (mask 2), the condition AP20A100, shows a significant decrease in PP compared to the conditions AP20A90RLarge (*P* < 0.001), AP11A100 (*P* < 0.001) AP6A100 (*P* = 0.030) and AP6A80 (*P* < 0.001). For the proximal region on the lateral side (mask 3), AP20A100 also shows a significant decrease compared to the conditions AP20A90RLarge (*P* < 0.001), AP11A100 (*P* < 0.001) and AP6A80 (*P* < 0.001). The conditions AP11A100 (*P* = 0.009) and AP6A80 *(P* < 0.001) are the two conditions that show a significant increase in PP compared to the control condition.

### 3.4 Medial and lateral side of midheel

For the medial midheel region (mask 4), AP11A80 shows the largest decrease in PP compared to other rocker conditions. This decrease in PP is significant compared to AP20A100 (*P* = 0.002). For the same region on the lateral side (mask 5), a similar trend is seen, in the effect of the rocker conditions on the PP, as for the medial midheel region. The same condition (AP11A80) shows a (largest) significant decrease in PP compared to AP6A80 (*P* = 0.003).

### 3.5 Medial and lateral side of distal heel

For the medial distal heel (mask 6), there is no significant decrease in PP between the rocker conditions or compared to the control condition. On the lateral side (mask 7), the same is seen as for the medial side, where no significant decrease in PP is found between the rocker conditions or compared to the control condition. Only increases in PP are seen, with AP20A90RLarge showing the largest increase compared to other rocker conditions. This increase in PP is significant compared to the conditions AP20A90RSmall (*P* = 0.024), AP6A90 (*P* = 0.019) and AP11A100 (*P* = 0.002), where the latter condition shows the smallest increase in PP.

## 4 Discussion

The aim of this study was to analyze the effects of different heel rocker designs, configurated with different rocker radii, apex positions and apex angles, on the heel plantar pressure distribution. Selecting optimal heel rocker parameters, can help to mitigate the increase in PP in the heel, when (forefoot) rocker shoes are used to decrease the PP in regions such as the forefoot, extending beyond just the heel. The findings of this study revealed that in none of the heel regions significant decreases in PP compared to the control shoe were found. Contrary, in the proximal lateral heel (mask 3) two conditions, with one having a proximal apex position with a small apex angle (AP6A80) and the other a mid apex position with a large apex angle (A11A100), were found to significantly increase PP. There were no significant differences between these two conditions.

Although there was no significant reduction in PP in the proximal heel region (mask 1–3) between the experimental (rocker) and the control condition, a significant difference in PP was found between the experimental conditions. Condition AP20A100, with a distal apex position, a small rocker radius and a large apex angle, showed the largest significant decrease in PP compared to conditions with more proximally located apex positions (no matter which apex angle is used; AP11A100, AP6A100, AP6A90, AP11A80, AP6A80). As hypothesized, a distal apex position with a small rocker radius (AP20A100), resulted in a PP reduction in the proximal heel region. This condition (AP20A100) has an apex angle that deviates from the neutral (90°). Given that in the forefoot, apex angle changes cause a ML shift in PP, we hypothesized that changes in the apex angle of the heel rocker would also cause this shift [13,15,17]. However, with the application of deviating apex angles in the heel region, this ML shift is not seen. AP20A100 with a large apex angle, was expected to reduce the pressure in the lateral heel region (e.g., proximal lateral heel, mask 2) and not the entire proximal heel region (mask 1–3). The toe-out foot progression angle (13° in adults) that people generally adopt during walking, may play a role in this result, since it causes the progression of the ground reaction to be perpendicular to the apex [24].

Contrary to the expectation, no condition reduced the PP effectively in the midheel (mask 4 and 5). Compared to the control shoe, condition AP11A80 showed the largest PP reduction. However, this reduction was less than 2 % and not significant compared to the control shoe. Condition AP11A80, showed only a significant reduction in PP compared to some of the worst performing conditions (AP20A100 and AP6A80). In order to reduce the PP in the midheel, other solutions than or in addition to using rocker shoes, need to be taken into consideration.

For the distal heel, although not significant, all conditions showed an increase in PP compared to the control shoe. This result may be alarming, however since the average absolute PP values in the distal heel region are 98 kPa (medial) and 119 kPa (lateral) in the control shoe and still far below 200 kPa in the experimental conditions, it is not expected that the threshold of 200 kPa will be exceeded, if persons with DM and LOPS were to have the same pressure values. Even in the worst performing condition, AP20A100 (which is the best performing one for the proximal heel), the PP was only 128 kPa (lateral) and 110 kPa (medial), with both values well below 200 kPa. On the other hand, when a person with DM and LOPS shows pressures above 200 kPa in this distal heel region, heel rockers cannot be adopted to reduce pressures in this region. In line with the midheel region, other interventions to reduce pressure have to be chosen.

This is the first study that systematically analyzes different heel rocker parameters on the heel plantar pressure. As a result, even with a restricted number of experimental conditions (not all combinations of a large radius with different apex positions and apex angles were tested with different rocker radii), valuable information is provided on the selection of appropriate heel rocker parameters. This information is particularly relevant when considering forefoot rocker shoes as an intervention to decrease the PP in regions other than the heel among persons with DM and LOPS. The effectiveness of forefoot rockers on pressure in the forefoot region has been proven in different studies [13,14,17]. However with increase in heel pressure as a negative side effect [13,14,17]. This study provides information how to minimize these side effects. Besides, if forefoot rocker shoes are adopted to reduce PP in regions other than the heel, additional solutions such as custom-made insoles for instance might be needed to effectively reduce the PP in the heel. Even though the reduction in PP may not be optimal, using a rocker shoe with a distal heel apex position, a small rocker radius and a large apex (100°) angle can help minimize the PP in the proximal heel region. As only young healthy adults were included in this study, the results found might not translate directly to persons with DM. However, Chapman et al. showed, despite differences in the plantar pressure, similar results in the effect of the rocker parameters on PP between persons with DM and healthy adults [17]. Lastly, there was no accommodation period, nonetheless the measurements started when the participants established a stable gait pattern in order to measure the immediate effects.

The rocker midsole used in this study is made out of rigid material (Polyamide 12; 80 durometers, shore D), that proofed to be effective in reducing the high PP values in the forefoot [14], yet the same material in the heel region results in poor shock absorption that likely increases the plantar pressure during the loading response. Therefore, it would be interesting to analyze in future studies whether more compliant mid- or outsole materials, padding with soft materials and/or insoles (in combination with rocker shoes) can decrease the high PP values in the heel region.

Although we focused in our study on PP relief of different heel rocker shoe designs, through in-shoe pressure measurement in persons with DM and LOPS, the pressure measurement technology does not provide in-depth information on mechanical changes such as strain distribution and internal stress of the foot structure and joint contact pressure. This information can be provided through computational modelling such as finite element modelling (FEM), which has been also used in footwear optimization intended to increase athletic performance and decrease the risk of sport related injuries [25,26]. Future studies can focus on FEM for an in-depth understanding of the footwear optimization and footwear biomechanics.

## 5 Conclusion

In conclusion, the current study provides initial evidence that when using rocker shoes as an intervention to reduce the plantar pressure in the forefoot, a heel rocker midsole with a distal apex position, a small rocker radius and an apex angle of 100°, mitigates the negative effects on plantar pressure in the proximal heel region. As with these rocker parameters the plantar pressure is increased in the medial and distal heel, the same heel rocker should not be used when pressures are approaching or exceeding 200 kPa in the midheel and distal heel region. For the midheel and distal heel, no rocker shoe applied in the current study reduced the PP more than 2 % compared to the control shoe. Other footwear options such as insoles should be adopted if reducing the PP in these regions is required as an addition to or instead of rocker shoes.

## Data availability

The anonymized data that support the findings of this study are available on DataverseNL with the following digital object identifier (10.13039/100000201DOI): https://doi.org/10.34894/4CG3ZE.

## CRediT authorship contribution statement

**Athra Malki:** Conceptualization, Data curation, Formal analysis, Software, Visualization, Writing – original draft, Writing – review & editing. **Laurens van Kouwenhove:** Conceptualization, Data curation, Investigation, Methodology, Software, Writing – review & editing. **Gijsbertus J. Verkerke:** Conceptualization, Formal analysis, Supervision, Visualization, Writing – review & editing. **Rienk Dekker:** Conceptualization, Formal analysis, Supervision, Visualization, Writing – review & editing. **Juha M. Hijmans:** Conceptualization, Formal analysis, Methodology, Supervision, Visualization, Writing – review & editing.

## Declaration of competing interest

The authors declare that they have no known competing financial interests or personal relationships that could have appeared to influence the work reported in this paper.
